# The Potential Correlation Between Nature Engagement in Middle Childhood Years and College Undergraduates’ Nature Engagement, Proenvironmental Attitudes, and Stress

**DOI:** 10.3389/fpsyg.2020.540872

**Published:** 2020-10-29

**Authors:** Naomi A. Sachs, Donald A. Rakow, Mardelle McCuskey Shepley, Kati Peditto

**Affiliations:** ^1^Department of Plant Science and Landscape Architecture, University of Maryland, College Park, MD, United States; ^2^Section of Horticulture, School of Integrative Plant Science, Cornell University, Ithaca, NY, United States; ^3^Department of Design and Environmental Analysis, Cornell University, Ithaca, NY, United States; ^4^Department of Behavioral Sciences and Leadership, United States Air Force Academy, Colorado Springs, CO, United States

**Keywords:** middle childhood, college students, university students, nature engagement, mental health, stress, environmental stewardship, pro-environment attitudes

## Abstract

**Introduction:**

Nature engagement (NE) provides myriad psychological and physiological benefits, many of which begin in childhood and continue into adulthood. Research suggests children who have positive experiences with nature are more likely to continue engaging with nature and have more proenvironmental attitudes (PEAs) as adults. Among the benefits of NE are reduced stress, improved sleep, and improved cognitive performance, all essential criteria for healthy undergraduate life. College students in particular, because of high levels of stress, may benefit from NE, and the frequency and type of their engagement may be impacted by childhood experience.

**Objective:**

This study aimed to better understand the potential correlation between university undergraduates’ past NE in their middle childhood years (MCYs) and current NE; past NE and undergraduate PEA; and undergraduate NE and stress levels. We chose to examine the middle childhood and undergraduate years because little research has been conducted on the relationship of NE between these two age groups.

**Methods:**

We used a survey of undergraduate students (*n* = 309) enrolled at a US university to explore the frequency and types of NE during MCYs, their family and neighborhood demographics, and current levels of NE, PEA, and stress in their undergraduate lives.

**Results:**

Although results indicated a large decrease in NE from middle childhood to undergraduate years for most participants, we found a significant positive correlation between NE during MCYs and undergraduate NE. We found a positive correlation between MCYs NE and undergraduate PEA as well as undergraduate NE and undergraduate PEA. Contrary to other studies and to our hypothesis, we did not find a correlation between undergraduate NE and reduced stress levels.

**Conclusion:**

This study looked specifically at US undergraduate students to compare their current engagement with and attitudes toward nature and the environment with their nature experiences during their formative MCYs. Our results suggest that it is important for people to have positive experiences with nature in childhood, both for continued NE and to inculcate PEAs in adulthood. These results can help in formulating approaches to improving student well-being at institutions of higher learning.

## Introduction

### Physiological and Psychological Benefits of Nature Engagement

Nature engagement (NE) can positively affect overall well-being by reducing stress and anxiety (Frumkin et al., 2017; Markevych et al., 2017; Bratman et al., 2019; Meredith et al., 2020), improving concentration and recall (Strife and Downey, 2009; Bratman et al., 2015), improving sleep patterns (Grigsby-Toussaint et al., 2015), and improving mood and outlook (Ulrich et al., 1991; Kaplan, 1995; Berman et al., 2008; Roe and Aspinall, 2011; Bratman et al., 2012; Brooks et al., 2017). For the purposes of this study, we define NE as *interaction with the natural world and all of its elements* (Zuo et al., 2016). As Zuo et al. (2016) describe, people engage with nature at different levels, from the more passive (looking at or sitting in a natural setting) to the more active (walking in a park or working in a garden). All types and levels of NE can be beneficial to health and well-being.

Various theories have been proposed for the physiological processes underlying these positive effects. Kuo (2015) argued that the mechanism by which NE affects physiological changes may be based on the stimulation of natural killer cells that are associated with boosting immune function. Bratman et al. (2015) identified the basis for changes in mental health to be associated with reduced neural activity in the subgenual prefrontal cortex, resulting in decreased levels of self-reported rumination and improved ability to concentrate.

While engagement with nature provides benefits regardless of age, many researchers are increasingly focusing on the relationship between children and nature. Studies have reported that simply spending time in nature can reduce children’s stress levels and improve well-being. Researchers in Denmark found that higher levels of greenness in children’s residential neighborhoods are negatively correlated with the likelihood of developing mental health problems later in life (Engemann et al., 2019). Similarly, Wells and Evans (2003) found that among a cohort of rural elementary-aged youth, the impact of life stresses was lower among children with high levels of nearby nature than among those with little nature nearby.

For children, NE can also affect behavioral indicators. In a recent systematic review of literature assessing the relationship between NE and mental health in children and teenagers, Tillmann et al. (2018) found that such engagement positively affected children’s emotional well-being, decreased attention deficit disorder and attention-deficit/hyperactivity disorder symptoms, and improved overall mental health. A Dutch study of two meta-analyses of existing literature found a positive correlation between NE and self-regulation in children (Weeland et al., 2019).

### Nature Engagement and Developmental Theories

Piaget (1962) described four stages of child development, from infancy through adolescence. Of these, the third stage, Concrete Operations (middle childhood, approximately ages 7–11 years), occurs when a child’s thought processes become more mature and logic-based, which allows for greater exploration of the child’s environment. Youth at this stage are integrating themselves into both human and natural systems and are making sense of their place in relation to these constructs. As they identify, name, and classify organisms and non-living natural objects, children in the Concrete Operations stage are also enhancing their ability to sort and retain information and ideas (Kellert, 2002). For this study, we chose to focus on this third/Concrete Operations phase because of the importance of this stage in a child’s developing relationship with the outside world.

In contrast to Piaget, Erikson (1962) theorized that there are eight developmental stages between birth and age 18 years and categorized the middle childhood years (MCYs) as a stage of conflict between industry (competence) and inferiority (failure). Based on Erikson’s theory, McLeod (2018) has posited that as children cope with new learning and social demands, they may recognize their developing relationship with the natural world as a core competency, defined as the healthy balance between adequacy and doubt.

### Access to Nature, NE, and Proenvironmental Attitudes in Middle Childhood Years

For the purposes of this study, we have used the term “proenvironmental attitudes” (PEAs), which we define as *concern for the natural environment.* PEAs are viewed as precursors to “proenvironmental behavior,” “environmentalism,” and “environmental stewardship” (Wells and Lekies, 2006; Chawla, 2007; Evans et al., 2018).

In their MCYs, young people also become more social, developing peer bonds while remaining dependent on family, and become more confident in their explorations. It appears that unstructured time in nature may be more valuable than structured time at this life stage (Starling, 2011). According to Kellert (2002), children in MCYs are more likely to feel comfortable venturing into unfamiliar natural settings, expanding their knowledge and capacity to cope in these areas without adult supervision. As Chawla (2007) suggests, when children enjoy freedom to explore in nature, they are likely to have the most positive and meaningful experiences.

A number of researchers have found that parental attitudes toward nature impact the time that young children spend in nature (McFarland et al., 2014). Beginning at 7 years of age, children transition to an outward view of the world associated with increased empathy and morality (Mah and Ford-Jones, 2012), which is dependent on parental modeling (Schoeppe et al., 2017). Considerable research in recent years has also focused on children’s NE in their MCYs and how their adult models (parents, teachers, and so on) address attitudes toward the natural world. For example, Evans et al. (2018) found that children who grew up with mothers with more PEAs engaged in more proenvironmental behavior as young adults. Researchers in two other studies focused on environmentalists in Norway and Kentucky (Chawla, 2007) and adults in the United Kingdom, Greece, and Slovenia (Palmer et al., 1998) about the roots of their environmental activism. In both studies, participants’ two most frequent answers were that their activism was the result of positive experiences of natural areas in childhood and adolescence, and family role models.

In contradiction to these findings, researchers studying middle school students and their parents and teachers in North Carolina found that small class sizes and higher socioeconomic status (SES) translated to NE and PEA later in life, but that parents and role models did not affect later attitudes or behavior (Stevenson et al., 2014). Researchers in another study point out that those growing up in underresourced communities often have less access to and experience with the natural world, resulting in less development of PEAs in adulthood (Powell et al., 2004).

One should not automatically associate proximity to neighborhood nature in childhood with frequency of NE. Soga et al. (2016) found that when undergraduates at a university in Tokyo were surveyed, those students who had grown up in areas with larger amounts of natural greenness did not necessarily report higher nature relatedness. This finding suggests that an individual’s positive emotional affinity toward nature is not determined merely by the proximity of natural settings in their surroundings, but also by the frequency and quality of the NE.

Asah et al. (2018) studied the mechanisms through which children experience nature and the longer-term impacts of those experiences. They found that childhood NE on one’s own or with friends is strongly associated with both environmental stewardship and commitment to NE in adulthood. However, while childhood NE through family outings predicted adult NE, it was not predictive of adult environmental stewardship.

### Relationship Between Childhood, Adolescent, and Young Adult NE and PEAs

Researchers have revealed that both NE and PEAs tend to peak during the MCYs and start to decline in adolescence. In one study (Szagun and Mesenholl, 1993), 12-year-old participants showed higher concern for the environment (PEAs) than 15- and 18-year-olds, whereas in another study (Pol and Castrechini, 2013), 9- to 13-year-olds had the highest PEAs, beliefs, and behaviors of four different age groups.

As young people progress from MCYs through adolescence and into young adulthood, the experiences they had as children help to shape their attitudes and behaviors. Jensen and Olsen (2019) explored the relationship between NE in early childhood (prior to age 11 years) and proenvironmental decision-making as adults. They found that adult respondents who, as children, had participated in nature-related activities with their families at least once a week were more likely to support an expensive clean water initiative than were those who had less frequently engaged with nature. Similarly, Rosa et al. (2018) found that greater contact with nature during childhood was associated with greater NE as an adult, as well as being positively associated with proenvironmental behavior.

### Potential Importance of NE for Undergraduate College Students

The topic of the role of nature as children progress into young adulthood as college students is important because of the reports of increased stress, anxiety, and depression in university populations. Undergraduate students in the United States face a plethora of challenges and stress inducers, including financial constraints, academic pressure, social pressures in the age of social media, and grappling with various forms of harassment (Twenge, 2014; Prabhakararao, 2016). Within the 12 months prior to a 2019 survey, US college students reported more than average or tremendous stress (59%), feeling overwhelming anxiety (66%), and hopelessness (56%). Thirteen percent had been diagnosed or treated for depression or anxiety (American College Health Association, 2019). A survey of 139 colleges reported a 30% rise in appointments with counselors between 2009–2010 and 2014–2015, although school enrollments had grown only 5%. Of those students, 61% reported anxiety, 49% depression, and 45% stress (Winerman, 2017). Undergraduate college students could benefit significantly from non-pharmacological mental health treatment modalities such as NE (Meredith et al., 2020).

### Impact of MCY Level of Urbanism and Socioeconomic Status on NE and PEAs

Among the considerations in this study is the impact of children’s MCY physical environment on their later NE and PEAs as undergraduates. Access to nature is typically more limited in dense urban areas (Cox et al., 2017). Adults who spent childhood in heavily urbanized environments (high density of buildings) report lower PEAs in adulthood than those who grew up next to natural elements such as flowerbeds and parks (Lohr and Pearson-Mims, 2005). Additionally, access to nature is inequitably distributed across socioeconomic classes (Shanahan et al., 2014). Comparisons between neighborhoods of differing socioeconomic levels have found that children in low-income communities have reduced access to parks and even lower levels of access to parks with play amenities (Rigolon and Flohr, 2014; Oliphant et al., 2019).

### Hypotheses and Research Questions

In this study, we used a survey of undergraduate students enrolled at a northeastern US university to explore the association between participants’ NE in MCYs (ages 7–11 years) and their current level of NE. We also examined the role of participants’ current NE, PEAs, and stress level. We primarily used closed-ended questions for quantitative analysis but included two open-ended questions that would provide a more qualitative view of the study. Based on our literature review, we had four *a priori* hypotheses:

• H1. NE in MCYs is positively correlated with NE in undergraduate years.
• H2. NE in MCYs is positively correlated with PEAs in undergraduate years.
• H3. Undergraduate NE is positively correlated with PEAs in undergraduate years.
• H4. NE in undergraduate years is negatively correlated with undergraduate self-perceived stress.

We were also interested in how participants’ physical environment in MCYs was associated with their current degree of NE, which we explored with the following two research questions:

RQ1: perceived level of urbanization in MCYs will be negatively correlated with NE in those years, and RQ2: perceived socioeconomic status (SES) in MCYs will be positively correlated with NE in those years.

## Materials and Methods

### Survey Development and Content

Our literature review informed the structure and questions of the Undergraduate Attitudes Toward Nature Survey (Appendix/Supplementary Materials). We first developed the survey in Microsoft Word and then entered it into Qualtrics v3.18, an online survey management platform, for online distribution. This research received approval for exemption from the university’s institutional review board in fall 2017.

The survey consisted of 24 questions in three parts: demographics, MCYs, and undergraduate (Table 1). The first part asked participants two demographic questions (gender and race/ethnicity). In the second section, five questions addressed the participants’ physical environment in their MCYs and included six questions about their level and type of NE. In the third section, nine questions focused on participants’ current life as undergraduate students, including what activities they engage in (NE), their concern for the environment (PEAs), and their stress level.

**TABLE 1:** Undergraduate nature engagement questionnaire.

Demographics—general
**Q1. With what gender do you identify?**
Male | Female | Additional gender category | I prefer not to say
**Q2. With what group do you identify?**
White | Hispanic, Latinx, or Spanish Origin | Black or African American | American Indian or Alaska Native | Asian | Native Hawaiian or Pacific Islander | Middle Eastern or North African | Biracial or multiracial | I prefer not to say

**Middle childhood**

**Q3. During your middle childhood years (ages 7–11 years), did you reside in the continental US for at least 2 years?**
Yes | No
**Q4. If yes, in what US state did you reside for the longest period during those years?**
**Q6. Which of the following demographic descriptions best fits where you lived for the longest period during middle childhood?**
Urban area | Small city or village | Suburban | Rural
**Q7. During your middle childhood years, what was your perception of your family’s economic status?**
Upper class | Upper middle class | Middle class | Lower middle class | Working class
**Q8. When you think back to your middle childhood years, what is the physical environment in which you first picture yourself?**
**Q9. Which of the following nature experiences did you engage in during your middle childhood years? (select all that apply)**
Taking walks in nature | Visiting local parks | Going to the beach | Working on a farm | Helping with a home garden | Hunting and/or fishing | Working with/caring for animals | Other (please specify)
**Q10. During your middle childhood years, did you attend a camp that included nature-based activities?**
Yes | No
**Q11. During your middle childhood years, how frequently do you recall spending time in nature?**
Daily | 3–4 times a week | 1–2 times a week | Less than once a week | Almost never
**Q12. During your middle childhood years, how frequently do you recall adults in your life (parents, guardians, relatives, teachers) talking about nature or the natural environment?**
Daily | 3–4 times a week | 1–2 times a week | Less than once a week | Almost never
**Q13. In your middle childhood years, what were your three favorite indoor non–school-related activities? (select three)**
Organized sports | Reading | Playing video games | Hanging out with family or friends | Exercise | Artistic expression | Watching TV | Other (please specify)
**Q14. In your middle childhood years, what were your three favorite outdoor non–school-related activities? (select three)**
Organized sports | Reading | Being outside in nature | Hanging out with family or friends | Exercise | Artistic expression | Working with/caring for animals | Camping | Hunting and/or fishing | Other (please specify)

**Undergraduate**

**Q15. What is your current class year at Cornell?**
First year | Sophomore | Junior | Senior | Unspecified
**Q16. What is your area of study at Cornell?**
**Q17. When you are feeling stressed at school, in what ways do you seek relief? (select all that apply)**
Talking to friends or family | Using alcohol or drugs | Talking with a counselor | Being outside in nature | Going to parties | Creative expression | Exercising indoors | Exercising outdoors | Frequent eating | Social media | Meditation or prayer | Other (please specify)
**Q18. During the semester, how frequently do you take recreational walks in nature on campus?**
Daily | 3–4 times a week | 1–2 times a week | Less than once a week | Almost never
**Q19. During your time at Cornell, how many afternoon labs or other courses have you taken that involve spending time in nature?**
5 or more | 3 to 4 | 1 to 2 | None
**Q20. During your time at Cornell, have you heard of the NatureRx program?**
Yes | Not sure | No
**Q21. Among the many economic, social, and political issues in the US, how would you rank your concern for the environment?**
1 = the environment is not important, 10 = very important
**Q22. On a scale of 1 to 10, how would you describe your overall stress level during the semester?**
1 = the least stressed, 10 = most stressed
**Q23. Please provide any additional thoughts regarding your current relationship with nature.**

For the first six MCY questions, we asked whether participants had spent at least two of their MCYs in the United States. We focused on the United States to reduce confounding variables. If students answered in the negative, they were routed to the end of the survey. For the SES question, because we were more concerned with *perceived* economic status, we did not define categories by income. In addition, children—including those in high school—typically do not know their precise family income or other economic status (Anderson and Holt, 2017). The final question under the first cluster asked participants, “When you think back to ages 7–11 years, what is the physical environment in which you first picture yourself?” Previous researchers have noted that common early childhood memories are associated with outdoor environments (e.g., Sebba, 1991), and we were interested in exploring this concept qualitatively.

For the six MCY NE questions, we first asked participants to select the nature experiences they had engaged in during ages 7–11 years. Some activities were more recreation-oriented, such as “taking walks in nature” and “going to the beach,” whereas others were more “work”-oriented such as “working on a farm” or “helping with a home garden.” We asked whether participants had attended a nature-based camp, how frequently they recalled spending time in nature, and how frequently they recalled adults in their lives talking about nature. Finally, we asked participants to choose their three (out of nine) favorite indoor and then their three (out of 11) favorite outdoor activities.

The third part of the survey focused on participants’ life as undergraduate students: in addition to undergraduate level and major, we asked a multiple-choice question about ways students sought relief from stress during the semester. Four questions addressed participants’ current level of NE. One question asked students whether they were familiar with the university program that prescribes time in nature.

The final two closed-ended questions asked participants to rank on a 1- to 10-point scale (1) their level of concern for the environment (PEAs) and (2) their overall stress level during the semester. Lastly, we asked participants in an open-ended question to provide any additional thoughts on their current relationship with nature.

### Pilot Testing

We pilot tested the survey in fall 2017 with a class of approximately 50 undergraduate horticulture students. We distributed paper surveys during class and asked the students to answer the questions and write additional comments about clarity of wording and whether they felt anything was missing or redundant. We then facilitated a follow-up discussion and, as a result, made minor changes, for example, changing “Latino” to “Latinx” and replacing the term “middle childhood years” with the specific ages (7–11 years). The final online survey was sent to five people (students, faculty, and staff) to assess ease of response in Qualtrics format. These two steps enhanced the survey’s usability and content validity.

### Dissemination

Announcements about the survey were sent to all undergraduate students at this campus via an e-newsletter. Students 18 years or older were encouraged to participate with an incentive of “$1.00 off a cup of coffee or tea” from an on-campus café/restaurant. The first announcement was sent out on Monday, February 26, 2018. Reminders were sent on March 5, 7, 12, and 19. The survey was closed on March 30, 2018.

### Demographic Composition

#### Gender

The majority of survey participants identified as female (83.8%), followed by 15.3% of participants identifying as male, two participants selecting an “additional gender category/identity,” and one participant selecting “I prefer not to say” (Table 2). Compared to the undergraduate university population in fall 2017 (52% female), a substantially higher proportion of females completed this questionnaire. We found no statistical associations with these moderating variables.

**TABLE 2:** Frequency statistics by demographic variables.

Characteristic	*n*	%
**Gender**		
Male	47	15.3
Female	258	83.5
Additional gender category/identity	2	0.6
I prefer not to say	1	0.3
**Racial composition**		
White	185	51.4
Asian	100	27.8
Hispanic, Latinx, or Spanish Origin	38	10.6
Biracial or multiracial	15	4.2
Black or African American	12	3.3
Native Hawaiian or Pacific Islander	4	1.1
Middle Eastern or North African	3	0.8
American Indian or Alaska Native	1	0.3
I prefer not to say	1	0.3
None of these	1	0.3
**Childhood region—by climate (ages 7–11 years)**		
Northeast	173	61.0
West	31	11.0
Southeast	22	7.7
Central	14	5.0
Midwest	13	4.7
Northwest	12	4.3
South	10	3.6
Southwest	8	2.5
Plains and Rockies	1	0.4
**Level of urbanism—by development (ages 7–11 years)**		
Urban	70	22.7
Small city or village	40	13.0
Suburban	174	56.5
Rural	24	7.8
**Perceived childhood economic status (ages 7–11 years)**		
Upper class	14	4.6
Upper middle class	124	40.4
Middle class	128	41.7
Lower middle class	21	6.8
Working class	20	6.5

#### Race/Ethnicity

The majority of participants identified as white (51.4%), followed by Asian (27.8%), Hispanic, Latinx or Spanish (10.6%), biracial or multiracial (4.2%), and Black or African American (3.3%). This sample more closely mirrors the composition of the fall 2017 undergraduate population, although the proportion of white students in the university population is lower (38%). Underrepresented minority students (Black, Hispanic, American Indian, Hawaiian/Pacific Islander, or multiracial students) comprised 22% of the undergraduate population in 2017 and 20% of the survey respondents (Table 2).

#### MCY Environment

The majority of participants had lived in the United States for at least 2 years during ages 7–11 years; 22 participants (7%) had not, and these 22 questionnaires were excluded from statistical analysis. Thirty-five states and the District of Columbia were represented. The highest proportion of participants (31.3%) reported having lived primarily in New York during MCYs. New Jersey and California were each represented by 30 participants (10.6%). This reflects the undergraduate university population, in which most students are from New York State (25%), the Middle States (MD, PA, NJ, DE, DC; 13%), and the West (11%). Table 2 reflects the composition of participants by NOAA Climate Region.

When considering the population and SES of their childhood environment, most participants represented suburban and urban areas (56.5 and 22.7%, respectively), and only 7.8% of students were from rural areas. Most participants perceived their family’s SES during MCYs as either middle class (41.7%) or upper middle class (40.1%).

A χ^2^ goodness-of-fit test suggests that suburban students who perceive themselves to be upper or upper middle class are overrepresented in this sample, whereas urban students of upper or upper middle class are underrepresented, as are suburban students of lower-middle and working class. Although not representative of an evenly distributed population, the sample is relatively representative of the university population in which the average family income of students is in the 79th percentile nationally, and nearly two-thirds of students are from the top 20% nationally in family income. Only 3.8% of students at this university are from the bottom 20% of family income nationally (citation Cornell University, 2020).

### Analytic Approach

A total of 362 Qualtrics surveys were filled out. Surveys less than 90% complete and that had taken fewer than 118 seconds to complete were omitted, resulting in a total of 309 surveys used for statistical analysis. Quantitative data were analyzed using Statistical Package for Social Sciences (SPSS) v23. Qualitative data were analyzed using Atlas.ti v8.

#### Quantitative Methods

For analyzing binary and Likert scale questionnaire items, differences between groups were assessed with a *t*-test, using *p*-values with equal variances not assumed when necessary. For examining the correlation between two Likert scale items, we reported Kendall τ_b_, because this was appropriate for data sets with ties (Agresti, 2002). For correlating Likert scale items and MCY nature experiences (a sum), we similarly reported correlations with Kendall τ_b_.

To investigate the relationship between two categorical items, we employed a χ^2^ test of association, using adjusted standardized residuals to determine which combinations were overrepresented or underrepresented. When necessary, groups with small numbers were combined to meet assumptions of a χ^2^ test of association. In particular, for the SES item, we combined “upper class” with “upper middle class,” and combined “lower middle class” with “working class.” Bonferroni corrections to alpha levels were performed when multiple tests were conducted.

For H1, “nature engagement (NE) in MCYs is positively correlated with NE in undergraduate years,” *NE in MCYs* was operationalized through three questionnaire items:

Q10: At any time during ages 7–11 years, did you attend a camp that included nature-based activities?
Q11: During ages 7–11 years, how frequently do you recall spending time in nature?
Q12: During ages 7–11 years, how frequently do you recall adults in your life (parents, guardians, relatives, teachers) talking about nature or the natural environment?

*NE in undergraduate years* was operationalized through two questionnaire items:

Q18: During the semester, how frequently do you take recreational walks in nature on or off campus?
Q19: During the semester, how frequently do you engage in other nature-related activities (e.g., sailing, cycling, skiing, etc.)?

For H2 (“NE in MCYs is positively correlated with PEAs in undergraduate years”) and H3 (“Undergraduate NE is positively correlated with PEAs in undergraduate years”), *PEAs in undergraduate years* was operationalized with the questionnaire item:

Q21: Among the many economic, social, and political issues in the United States, how would you rank your concern for the environment? (1 = the environment is not important, 10 = the environment is very important)

For H4, “NE in undergraduate years is negatively correlated with undergraduate self-perceived stress,” *stress level* was operationalized with the following questionnaire item:

Q22: On a scale of 1–10, how would you describe your overall stress level during the semester? (1 = the least stressed, 10 = most stressed)

#### Qualitative Methods

Qualitative data collected during this study included answers to two open-ended survey items. In the MCYs section, “When you think back to ages 7–11 years, what is the physical environment in which you first picture yourself?” This question ties back to H1, which addresses NE in early childhood. In the undergraduate section, the final survey question was: “Please provide any additional thoughts regarding your current relationship with nature.” This allowed us to capture any additional qualitative data that might add context to the quantitative findings.

Three hundred thirty-nine participants (95%) provided a response to the first open-ended question. Most responses were short phrases or sentences, averaging 7.3 words per response. Following Saldaña (2013), a member of the research team identified codes from the data and organized those codes into eight categories (Figure 2). One hundred fifty-four participants (43%) responded to the final/second open-ended question. Because of the smaller number of responses, we did not conduct the Saldaña method of qualitative analysis on this question.

## Results

### Relationship Between NE in Middle Child Years and NE in Undergraduate Years (H1)

**FIGURE 1:**
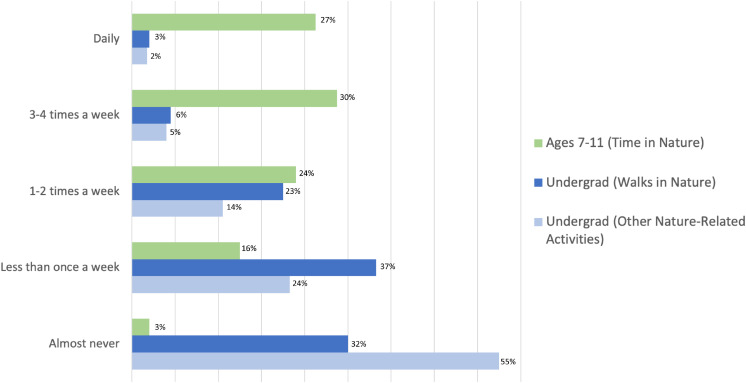
Frequency of nature engagement decreases from childhood to undergraduate years. Supporting H1, participants who engaged with nature (NE) more frequently in MCYs engaged with nature more frequently as undergraduates (recreational walks, τ_b_ = 0.223, *p* < 0.001, and other nature-related activities as undergraduates, τ_b_ = 0.306, *p* < 0.001). *Post hoc* tests revealed that participants who engaged with nature daily in MCYs were more likely to engage with nature as undergraduates [take nature walks or engage in other nature-related activities one to two times a week (adjusted *R* = 3.1 and 4.3, respectively)].

There was a notable decrease in NE from MCYs to college years. The majority of participants engaged with nature during MCYs at least three to four times a week, although most undergraduates reported current NE as less than once a week (Figure 1).

**FIGURE 2:**
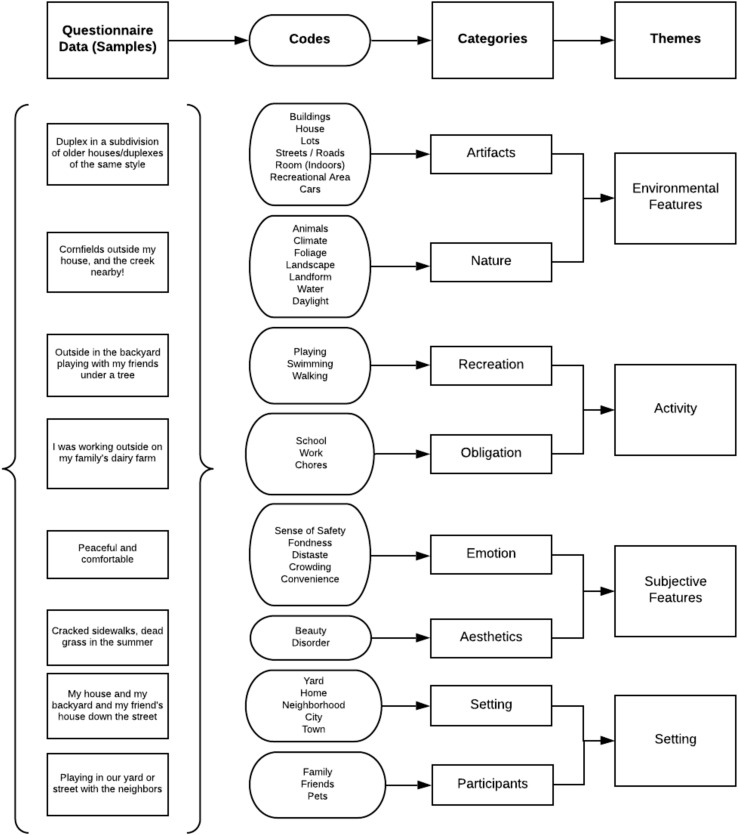
Qualitative elements of the childhood nature experience.

Similarly, undergraduates who recalled adults frequently talking about nature reported greater NE now (recreational walks, τ_b_ = 0.157, *p* = 0.001, other nature activities, τ_b_ = 0.214, *p* < 0.001). Although attending a nature camp was not significantly correlated with frequency of nature walks now, *t*(305) = −0.90, *p* = 0.369, there was a significant correlation between camp attendance and frequency of other undergraduate nature activities: participants who went to camp in MCYs engaged in other nature activities as undergraduates more frequently (mean = 4.49) than those who did not attend camp (mean = 4.12), *t*(222.9) = −3.14, *p* = 0.002.

The qualitative question regarding early memories of nature provided more detail on NE in MCYs. Within the eight categories identified during the analysis, four themes emerged: environmental features (artifacts and nature), activity (recreation and obligation), subjective features (emotion and aesthetics), and setting (physical setting and participants). This relationship is represented in Figure 2.

Environmental features included physical characteristics of both the built and natural environment, for example, “Cornfields outside my house, and the creek nearby!” Subjective features were emotional or aesthetic, such as this statement by a participant: “Peaceful and comfortable.” References to activities addressed both recreation and obligation, like “I was working outside of my family’s dairy farm.” Statements related to the setting included the setting itself and the participants, such as the case in this quote: “Playing in our yard or street with neighbors.”

These themes represent components of the childhood nature experience, including environmental features, subjective features, activity, and setting. When viewed through the lens of the childhood physical environment (rural, suburban, small city, urban), the quality of the childhood experience depends somewhat on the level of development in a participant’s community. Survey participants from rural settings more frequently described the natural features of their MCY environments, with the number of references to artificial features increasing as the environment was increasingly urbanized.

### Relationship Between NE in MCYs and PEAs in Undergraduate Years (H2)

Supporting H2, there was a positive correlation between participants who engaged more with nature in MCYs and undergraduate PEAs, τ_b_ = 0.169, *p* < 0.001. Results from the survey also suggest children who heard adults talk about nature more frequently ranked higher with PEAs, τ_b_ = 0.158, *p* = 0.001. Camp attendance in MCYs, however, was not correlated with increased PEA, *t*(305) = 0.41, *p* = 0.68.

### Relationship Between Undergraduate NE and Undergraduate PEAs (H3)

Supporting H3, participants who engaged with nature as undergraduates reported greater PEAs (walked more in nature, τ_b_ = 0.139, *p* = 0.003, and engaged in other nature-related activities, τ_b_ = 0.132, *p* = 0.007).

### Relationship Between Undergraduate NE and Undergraduate Stress Level (H4)

**FIGURE 3:**
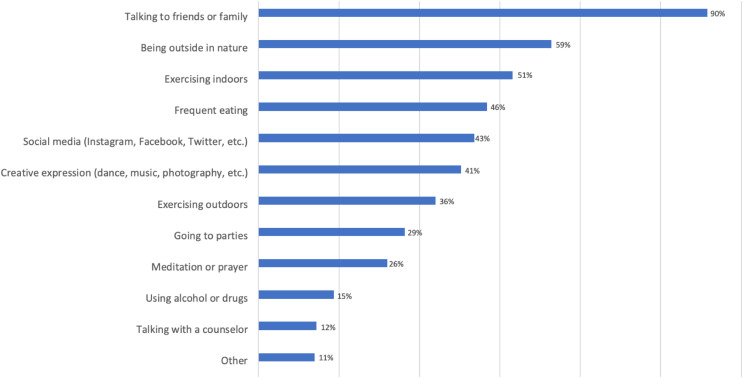
Stress relief strategies and activities (undergraduate).

Students described the strategies and activities they engaged in to relieve stress (Figure 3). Being outside in nature was exceeded only by talking to friends or family. Nevertheless, contrary to H4, we found no significant correlation with participants’ NE (walk more in nature or engage in other nature-related activities) and self-reported stress level, *p* > 0.05.

The final open-ended question provided insight regarding the lack of current NE and, perhaps, the lack of correlation with stress levels. In 34 of the open-ended responses, students said that they “wished” they could spend more time in nature or “should” spend more time in nature. The two largest reported barriers were time (*n* = 35) and weather (*n* = 21). Other barriers included lack of transportation off campus, fear of insects, worry about sun damage, and stress itself (n = 11). As one student articulated, “Paradoxically, the more I would like to be in touch with nature (during periods of stress), the less able I am to actually explore it.”

Several students mentioned the beauty of the campus and surrounding environment as an initial draw and an asset during times of stress: (1) “I love it. It brings me joy and peace and comfort. A very significant factor in my choice to attend Cornell was it’s [*sic*] natural beauty and the availability of the forest both on campus and in nearby locations.” (2) “When I visited Cornell I knew it was the perfect place for me in part because of the extensive opportunities for outdoor activities in nature. However, I don’t spend enough time in nature during the semester, even though it has a great calming effect on me. I lazily resort to things I can access more easily, such as my computer, for stress relief.” (3) “I don’t have time to go outside purely to enjoy the outdoors, but I enjoy walking between classes and stargazing at night on my way home. Part of the reason I moved to Ithaca was to get away from the city and immerse myself in nature.”

### Perceived Levels of MCY Urbanization and Socioeconomic Status and NE (RQ1, RQ2)

As suggested by RQ1, there was a negative relationship between residing in an urban environment and NE in MCYs. Participants who reported frequent MCY NE tended to be from more rural communities, τ_b_ = −0.27, *p* < 0.001. *Post hoc*-adjusted residuals suggest participants from rural areas were most likely to engage with nature daily in MCYs (adjusted *R* = 5.5), whereas participants from urban areas were most likely to engage with nature less than once a week (adjusted *R* = 4.6). Participants who attended a nature camp grew up in a more rural environment (mean = 2.57) than participants who did not attend a nature camp (mean = 2.33), *t*(179.786) = −3.14, *p* = 0.041. Participants who recalled adults frequently talking about nature in MCYs tended to be from more rural communities, τ_b_ = 0.153, *p* = 0.002.

Participants who went to a nature camp grew up in a higher socioeconomic class (mean = 2.58) than those who did not go to a nature camp (mean = 2.96), *t*(304) = −3.50, *p* = 0.001. Participants who recalled adults frequently talking about nature also tended to be from a higher SES, τ_b_ = 0.118, *p* = 0.015. There was not, however, a significant correlation between SES and NE in MCYs, τ_b_ = 0.087, *p* = 0.075 (RQ2).

In summary, contrary to RQ1, there was no significant negative correlation between MCY residency in an urban setting and current undergraduate NE as reported in frequency of nature walks, τ_b_ = −0.037, *p* = 0.451, or current frequency of other nature-related activities, τ_b_ = −0.067, *p* = 0.181. Likewise, there was no significant correlation between MCY SES and frequency of current recreational nature walks (RQ2).

## Discussion

### Relationship Between NE in Middle Child Years and NE in Undergraduate Years (H1)

Although we found a significant positive correlation between NE during MCYs and NE during undergraduate years, *post hoc* tests revealed a large decrease in NE from MCYs to undergraduate for most participants. The majority of participants engaged with nature during MCYs 3–4 times a week, whereas the majority of participants reported that they now spend time in nature less than once a week. Participation in outdoor activity reliably changes across the lifespan, revealing a demonstrated decline between childhood, adolescence, and young adulthood (Larson et al., 2011).

### Relationship Between NE in MCYs and PEAs in Undergraduate Years (H2)

Results suggesting a positive correlation between childhood NE and PEAs in undergraduate years are supported by existing literature. Wells and Lekies (2006) suggest “wild” activities during childhood, including hiking and camping, are positively associated with both adult NE and PEAs. Just as Wells and Lekies (2006) differentiate between “wild” and “domestic” outdoor activities, Ewert et al. (2005) also found a difference in PEAs in university students, depending on the frequency of “consumptive” or “appreciative” outdoor activities during childhood. Although the current study simply asked about walks in nature or other nature-related activities, future research in predictors of college student behaviors may consider differentiating between types of NE.

### Relationship Between Undergraduate NE and Undergraduate PEAs (H3)

Even though survey participants engaged with nature much less as undergraduates than they had in MCYs, we nevertheless found a positive correlation between NE as undergraduates and PEAs. This finding is consistent with previous research (Palmer et al., 1998; Chawla, 2007; Rosa and Collado, 2019; Alcock et al., 2020; Whitburn et al., 2020).

### Relationship Between Undergraduate NE and Stress (H4)

Although the findings from previous research support the restorative properties of NE regarding stress reduction (e.g., Frumkin et al., 2017; Markevych et al., 2017; Bratman et al., 2019; Meredith et al., 2020), results from the current study did not suggest a significant correlation with stress in either direction. This finding was particularly interesting because participants overall ranked “being outside in nature” (a marker of NE) second only to “talking to friends or family” as a way to relieve stress. This suggests some recognition of the restorative value of NE. Nevertheless, NE may be yet another healthy and/or pleasurable behavior that, like adequate sleep, proper nutrition, exercise, and so forth, falls by the wayside during stressful times (Weidner et al., 1996; Britz and Pappas, 2010).

Qualitative responses shed light on the discrepancy and lack of correlation. Some participants reported that they did not see spending time in nature as a “productive” use of their time: “I find that I enjoy nature a lot more when I am unstressed and have a lot of time to relax. I have tried to go hiking or go on walks when I am moderately stressed, but I tend to get too anxious about spending my time unproductively.”

Other possible explanations for this outcome include the potential lack of construct validity by using a single questionnaire item to measure stress. Furthermore, the overall high level of stress associated with college student life may have rendered the Likert scale ineffective. A high level of stress might be accepted as normal by many students (Winerman, 2017; American College Health Association, 2019). Future research may consider using a validated measure (perhaps specific to college students) to evaluate stress.

### Perceived Level of MCY Urbanization and Socioeconomic Status and NE (RQ1, RQ2)

We found evidence of a relationship between MCY SES, level of urbanization, and MCY NE, such that upper-SES participants reported more NE during MCYs, as did participants from more rural settings. Researchers have found that urban versus rural daily experience impact children’s concepts of nature (Collado et al., 2016). These findings did not extend, however, to NE as undergraduates. The intense challenges of university life and efforts toward adaptation may supersede normal behaviors and familial culture. Lifestyle changes have been recorded in freshmen students (Wolf and Kissling, 1984).

### Limitations

The survey was administered in the winter of 2018. A better time for distribution would have been in the middle of fall semester when the weather and daylight hours are more conducive to outdoor activities, and before end-of-semester stress inhibits survey participation. It is likely that participants’ NE was negatively influenced by the cold weather and short days. In fact, many responses to the final open-ended question mentioned the cold weather as a barrier to NE.

The majority of participants were first-year undergraduates (second-semester freshmen) who may have had less knowledge about where and how to access nature on or off campus. A larger sample of students who had lived in the area longer might have shown a higher level of NE.

Survey participants were a convenience sample of students who opened a community newsletter, saw the survey announcement, and clicked on the link. It is possible that students who were interested in the topic of “nature” were more likely to take the survey. A larger sample size with randomized responses would be ideal.

Results may not be generalizable to other universities. The site for this study was in a cool temperate climate and a rural community with significant access to nature amenities. However, the survey could be administered at other universities in the future. It would be particularly interesting to compare students at 4-year state schools where tuition is lower; compare areas of the country where climate and weather make nature more accessible year-round; and examine the responses of students in more urbanized areas such as New York City, Miami, or Chicago.

## Conclusion

This study surveyed undergraduate college students at a US university to compare their current engagement with and attitudes about nature with their nature experiences during their formative MCYs. The study confirmed what many other researchers in the United States and internationally have found—that parents or other adult figures who speak about the benefits of time in nature influence children to spend more time outdoors, and that such young people are also more likely to engage with nature as undergraduates than those whose parents less frequently spoke about nature in their MCYs.

Our findings implicate family and peers as important influences in the child’s life. The childhood environment is a complex, nested system in which community-level and neighborhood-level interactions impact children (Booth and Crouter, 2001), as well as family-level influences as a child constructs their future engagements and attitudes. In addition, participants from wealthier backgrounds and those from rural settings were more likely to have engaged with nature in MCYs than those from more urban settings or lower SES.

Our findings reinforce the importance of positive nature exposure and engagement in childhood. Whether “working” in nature on a farm or in a home garden, or engaging in more leisure-oriented activities such as going for walks or to the beach, these positive nature experiences may positively impact NE and PEAs in young adulthood.

An unexpected finding is how much less time undergraduate participants in this survey spend in nature currently than in their MCYs. Coupled with the lack of a positive correlation between NE and self-reported stress, one could conclude that undergraduates do not perceive NE as a viable approach to lowering their stress levels. This conclusion is belied by the fact that participants listed “being outside in nature” as the second most important means of reducing stress in their lives and that many participants spoke longingly of nature (wishing they had more time to spend in it or that the weather was more conducive) in their open-ended responses.

A likely explanation for this discrepancy is that students typically face very high levels of stress on a daily basis and that time in nature alone does not eliminate stress. A number of university counseling centers currently offer “nature prescriptions” as one of several tools that can be used to improve a student’s well-being. We recommend that, in doing so, counselors integrate the benefits of NE with other components of overall well-being, including a proper diet, adequate sleep, and healthy socializing. It will also be important to educate students on how to connect with “nearby nature” on campus safely and comfortably in all seasons.

While freshmen did not access nature as frequently as predicted, they indicated the desire to do so. An implication of this study is that engagement with nature should be more thoroughly integrated into freshmen orientation protocols, as well as a prescription proferred by university health clinicians. Outdoor classes, buildings designed with views of nature, and the introduction of indoor plants are other tools to facilitate NE.

Achieving a better understanding of how environmental, familial, and experiential factors from MCYs affect the nature engagement and PEAs of currently enrolled undergraduates can assist college and university counselors to anticipate problematic behavior in individuals before it arises. Given the enormous demands on campus counseling centers, such knowledge could improve the effectiveness of the services provided to undergraduates.

## Data Availability Statement

The datasets generated for this study are available on request to the corresponding author.

## Ethics Statement

The studies involving human participants were reviewed and approved by Cornell University Institutional Review Board. The patients/participants provided their written informed consent to participate in this study.

## Author Contributions

NS and DR: conceptualization, methodology, investigation, writing – original draft, and writing – review and editing. MS: conceptualization, methodology, writing – original draft, writing – review and editing, and supervision. KP: formal analysis, visualization, writing – original draft, and writing – review and editing. All authors contributed to the article and approved the submitted version.

## Conflict of Interest

The authors declare that the research was conducted in the absence of any commercial or financial relationships that could be construed as a potential conflict of interest.
