# Supplementary material for: The Potential Correlation Between Nature Engagement in Middle Childhood Years and College Undergraduates’ Nature Engagement, Proenvironmental Attitudes, and Stress
Source: Front Psychol. 2020 Oct 29;11:540872. doi: 10.3389/fpsyg.2020.540872 (PMC7658602; doi:10.3389/fpsyg.2020.540872)
Supplement: Supplementary file 1 [file Data_Sheet_1.PDF]

Introduction

.

Welcome to the [redacted] Undergraduate Attitudes Toward Nature Survey!
Your input is very important to us. This survey is completely anonymous. No information you share will be identified with your name or net id.

You must be 18 years or older to participate.

After you complete the survey, you will be eligible for a coupon for $1.00 off a cup of coffee or tea at [redacted].

Please review the Informed Consent Document below. By starting the survey, you are giving consent to participate.

. Informed Consent Document

Project Title:
Undergraduate Attitudes Toward Nature

Principal Investigator:
[redacted]

What the study is about
The researchers have developed a survey about [redacted] undergraduate students' attitudes toward nature, and are seeking participation from all [redacted] undergraduates 18 years and older.

What we will ask you to do

You have been asked to participate in this anonymous survey because you are an undergraduate student at [redacted]. The majority of the questions will be closed-ended, and the survey will take approximately 10 minutes.

Risks and discomforts

We do not anticipate any risks from participating in this research.

Benefits

There are no direct benefits to you as a participant. Information from this study may indirectly benefit the [redacted] community, the broader academic community, and society in general by adding knowledge to the field.

Payment for participation

Each participant who completes the survey will receive a coupon for $1.00 off a cup of coffee or tea at [redacted].

Privacy/Confidentiality

We anticipate that your participation in this survey presents no greater risk to privacy than everyday use of the Internet. Basic demographic information will be gathered but participants will be anonymous.

Taking part is voluntary

Your participation is voluntary. You may refuse to participate before the study begins, discontinue at any time, or skip any questions that may make you feel uncomfortable, with no penalty to you.

If you have questions

The main researcher conducting this study is [redacted]. If you have any questions or concerns

regarding your rights as a participant in this study, you may contact the Institutional Review Board (IRB) for Human Participants at [redacted] You may also report your concerns or complaints anonymously through Ethicspoint online at [redacted]. Ethicspoint is an independent organization that serves as a liaison between the University and the person bringing the complaint so that anonymity can be ensured.

Please feel free to print this form for your records.

Early Childhood Years

.

To begin, we would like to learn a little about your background, *especially during your childhood years from ages 7 - 11*.

Q1. **With what gender do you identify? (Please select one)**

- ◯ Male
- ◯ Female
- ◯ Additional gender category/identity
- ◯ I prefer not to say

Q2. **With what group do you identify? (Select all that apply)**

- ☐ White
- ☐ Hispanic, Latinx, or Spanish Origin
- ☐ Black or African American
- ☐ American Indian or Alaska Native
- ☐ Asian
- ☐ Native Hawaiian or Pacific Islander
- ☐ Middle Eastern or North African
- ☐ Bi-racial or multi-racial
- ☐ I prefer not to say

Q3. **During ages 7 – 11 of your childhood, did you live in the Continental U.S. *for at least two years*?**

- ○ Yes
- ○ No

Q4. **If you answered “Yes” to Question #3, in what U.S. state did you live *for the longest period* during these years?** Please choose from the drop-down menu.

Q5. **Please enter your zip code from that time, if you remember it. If not, you can skip this question.**

Q6. **Which of the following demographic descriptions best fits where you lived for the longest period of time during ages 7 - 11 (select one):**

- ◯ **Urban area** (an overall population of greater than 50,000 people, with a density of residences, businesses, and services)
- ◯ **Small city or Village** (population below 50,000 with a mix of residences and small businesses)
- ◯ **Suburban** (lying outside an urban area, with typically single family homes)
- ◯ **Rural** (population below 5,000, with residences spread out and fewer businesses and services)

Q7. **During ages 7 – 11, what was your perception of your family's economic status (select one):**

- ◯ Upper class
- ◯ Upper middle class
- ◯ Middle class
- ◯ Lower middle class
- ◯ Working class

Q8. **When you think back to ages 7-11, what is the physical environment in which you first picture yourself?** ***Don't overthink this question - put down whatever pops into your head first.***

Q9. **Which of the following nature experiences did you engage in during ages 7 - 11 (select all that apply):**

- ☐ Taking walks in nature
- ☐ Visiting local parks
- ☐ Going to the beach
- ☐ Working on a farm
- ☐ Helping with a home garden (vegetables, fruit, or flowers)
- ☐ Hunting and/or fishing
- ☐ Working with/caring for animals
- ☐ Other (please specify): ______

Q10. **At any time during ages 7 - 11, did you attend a camp that included nature-based activities (as opposed to a camp that mostly took place indoors, such as computer)?**

- ○ Yes
- ○ No

Q11. **During ages 7 - 11, how frequently do you recall spending time in nature (select one):**

- ○ Daily
- ○ 3-4 times a week
- ○ 1-2 times a week
- ○ Less than once a week
- ○ Almost never

Q12. **During ages 7 - 11, how frequently do you recall adults in your life (parents, guardians, relatives, teachers) talking about nature or the natural environment (select one):**

- ○ Daily
- ○ 3-4 times a week
- ○ 1-2 times a week
- ○ Less than once a week
- ○ Almost never

Q13. **During ages 7 – 11, what were your *three* favorite *indoor* non-school related activities (select THREE):**

- ☐ Organized sports (for example basketball, hockey, gymnastics)
- ☐ Reading
- ☐ Playing video games
- ☐ Hanging out with family or friends
- ☐ Exercise (for example swimming, jogging, yoga)
- ☐ Artistic expression (for example drawing, dance, music)
- ☐ Watching TV
- ☐ Other (please specify): ____________

Q14. **During ages 7 – 11, what were your *three* favorite *outdoor* non-school related activities (select THREE):**

- ☐ Organized sports (soccer, football, softball, etc.)
- ☐ Reading
- ☐ Being outside in nature
- ☐ Hanging out with family or friends
- ☐ Exercise (swimming, jogging, cycling, etc.)
- ☐ Artistic expression (drawing, dance, music)
- ☐ Working with/caring for animals
- ☐ Camping
- ☐ Hunting and/or fishing
- ☐ Other (please specify): ______

.

Now we would like to learn a bit about you as a student at [redacted].

Q15. **What is your current class year at [redacted] (select one):**

- ○ First year
- ○ Sophomore
- ○ Junior
- ○ Senior
- ○ Unspecified

Q16. **What is your area of study at [redacted].** Please choose from the drop-down menu.

Q17. **When you are feeling stressed at school, in what ways do you seek relief (select all that apply):**

- ☐ Talking to friends or family
- ☐ Using alcohol or drugs
- ☐ Talking with a counselor
- ☐ Being outside in nature
- ☐ Going to parties
- ☐ Creative expression (dance, music, photography, etc.)
- ☐ Exercising indoors
- ☐ Exercising outdoors
- ☐ Frequent eating
- ☐ Social media (Instagram, Facebook, Twitter, etc.)
- ☐ Mediation or prayer
- ☐ Other (please specify):

Q18. **During the semester, how frequently do you take recreational walks in nature on or near campus?**

- ◯ Daily
- ◯ 3-4 times a week
- ◯ 1-2 times a week
- ◯ Less than once a week
- ◯ Almost never

Q19. **During your time at [redacted], how many courses have you taken that involve spending time in nature (through Fall 2017; do not include this semester. Each course counts for one):**

- ◯ 5 or more
- ◯ 3 - 4
- ◯ 1 - 2
- ◯ None

Q20. **During your time at [redacted], have you heard of the "Nature Rx[redacted]" program?**

- ◯ Yes
- ◯ Not sure
- ◯ No

Q21. **Among the many economic, social, and political issues in the U.S., how would you rank your concern for the environment (1 = The environment is not important, 10 = The environment is very important):**

The environment is not important — The environment is very important

1 2 3 4 5 6 6 7 8 9 10

Q22. **1. On a scale of 1 - 10, how would you describe your overall stress level during the semester (1 = Least stressed, 10 = Most stressed):**

Least stressed — Most stressed

1 2 3 4 5 6 7 8 9 10

Q23. **Please provide any additional thoughts regarding your current relationship with nature:**

Q24. **Would you be interested in participating in a follow-up interview about this topic?** If so, please copy this address and email [redacted] with subject line "Survey follow-up."

Thank you and code

. Thank you for taking this survey! Please click the next button to receive your coupon.

Powered by Qualtrics
